# Roles of Microglial Phagocytosis and Inflammatory Mediators in the Pathophysiology of Sleep Disorders

**DOI:** 10.3389/fncel.2017.00250

**Published:** 2017-08-30

**Authors:** Agnes Nadjar, Henna-Kaisa M. Wigren, Marie-Eve Tremblay

**Affiliations:** ^1^Nutrition et Neurobiologie Intégrée, UMR 1286, Institut National de la Recherche Agronomique Bordeaux, France; ^2^Nutrition et Neurobiologie Intégrée, UMR 1286, Bordeaux University Bordeaux, France; ^3^OptiNutriBrain International Associated Laboratory (NutriNeuro France-INAF Canada) Québec, QC, Canada; ^4^Faculty of Medicine/Medicum, University of Helsinki Helsinki, Finland; ^5^Axe Neurosciences, CRCHU de Québec-Université Laval Québec, QC, Canada; ^6^Département de médecine moléculaire, Université Laval Québec, QC, Canada

**Keywords:** microglia, sleep, wakefulness, disease models, animal, diseases

## Abstract

Sleep serves crucial learning and memory functions in both nervous and immune systems. Microglia are brain immune cells that actively maintain health through their crucial physiological roles exerted across the lifespan, including phagocytosis of cellular debris and orchestration of neuroinflammation. The past decade has witnessed an explosive growth of microglial research. Considering the recent developments in the field of microglia and sleep, we examine their possible impact on various pathological conditions associated with a gain, disruption, or loss of sleep in this focused mini-review. While there are extensive studies of microglial implication in a variety of neuropsychiatric and neurodegenerative diseases, less is known regarding their roles in sleep disorders. It is timely to stimulate new research in this emergent and rapidly growing field of investigation.

## Introduction

Humans spend approximately a third of their lives sleeping. Sleep is an involuntary process, required to sustain good mental and physical health. During sleep, the brain processes information, consolidates memories, and undergoes a number of maintenance processes that help its function upon waking (Graves et al., 2001; Abel et al., 2013; Tononi and Cirelli, 2014; Vorster and Born, 2015). Loss or restriction of sleep is associated with multiple detrimental consequences (Musiek and Holtzman, 2016; Pires et al., 2016).

Microglia are macrophages derived from the embryonic yolk-sac that permanently reside in the brain alongside neurons, astrocytes, and oligodendrocytes (Gomez Perdiguero et al., 2013). These cells are uniformly distributed and their ramified processes constantly survey the brain parenchyma during normal physiological conditions. Microglia respond to pathology in various manners—they enlarge their cell bodies, increase their mobility, and hyper-ramify or reduce their processes. The microglia field has recently undergone explosive growth, especially since microglial roles in the healthy brain are becoming uncovered (Tremblay et al., 2011; Sierra et al., 2014). These mononuclear phagocytes are now recognized as essential contributors to neuronal survival, synaptic pruning, and dendritic spine formation during development, as well as synaptic maintenance and plasticity, neurogenesis, learning, memory, and cognition into adulthood (Salter and Beggs, 2014; Hong and Stevens, 2016; Tay et al., 2016). As the brain's immune cells, microglia help defend against pathogens, and play pivotal roles in recovery from sickness and injury (Kettenmann et al., 2011; Tremblay and Sierra, 2014). Microglia phagocytose cellular debris and release pro- and anti-inflammatory cytokines, trophic factors, and various other molecular mediators, processes critical for adaptation of the brain to the ever-changing environment (Delpech et al., 2015; Shemer et al., 2015; Tian et al., 2017).

Microglia detect changes in homeostasis through their recognition of exogenous or endogenous danger signals, pathogen-associated molecular patterns (PAMPs; small molecular motifs conserved within a class of microbes that are recognized by cells of the innate immune system via pathogen recognition receptors) and danger-associated molecular patterns (DAMPs; host-derived motifs that also regulate the activation of pathogen recognition receptors and can be triggered by enhanced neuronal activity and psychological stress). Microglia respond by orchestrating neuroinflammation through their interactions with peripheral immune cells which invade the brain, especially if the blood-brain-barrier is compromised (notably occurs during chronic sleep restriction, He et al., 2014), as well as vascular cells, glial cells, and neurons (Xanthos and Sandkuhler, 2014). In some contexts, microglia perform adaptive immune functions and present antigens to T cells upon binding to MHC class II expressed on their surface (Greter et al., 2015). Their responses can be homeostatic, leading to adaptation, but also dysfunctional, contributing to, or causing pathology. Anti-inflammatory mechanisms can be triggered in parallel to terminate neuroinflammmation and reduce pathological outcomes (Xanthos and Sandkuhler, 2014).

Considering that sleep and microglia share essential homeostatic functions, we have focused this review to examine the roles of microglial phagocytosis and inflammatory mediators, among various brain regions, in the pathophysiology of sleep disorders (see Table 1 for a summary). Although the number of studies providing direct evidence is currently limited, we anticipate this field of investigation to expand in the near future, considering the exponential growth of microglial research (and increased availability of tools to study these cells specifically) in recent years. Since very interesting findings on the topic recently came out, we also hoped to stimulate further the interest toward microglia and sleep.

**Table 1:** Microglial functions and relevance to sleep disorders.

**Neuroinflammation**	**Experimental findings**	**Suggested implication**
	**Infection-induced sleep gain**	
Microglia are the main producers of cytokines in the central nervous system during inflammatory diseases.	IL-1 and TNFα are well-known to promote sleep in humans and in animal models (Krueger, 2008; Krueger et al., 2011; Zielinski et al., 2013; Krueger and Opp, 2016).	Pro-inflammatory cytokines may exert somnogenic effects by promoting microglial attraction to synapses (Karrer et al., 2015). TNFα induces the production of chemokines (CCL2, CCL7, and CXCL10) by neurons, which bind to corresponding receptors expressed by microglia and are known to promote microglial process extension (Karrer et al., 2015).
	**Recreational-drug induced sleep loss**	
	PET scans of chronic d-METH self-administrating individuals reveals increased binding of the radiotracer [11C](R)-PK11195 that labels “activated” microglia (McCoy et al., 2007).	
	In mice undergoing microglial depletion, the duration of daily wakefulness produced by d-METH is reduced by nearly 1 h. *Ex vivo* nitric oxide synthase (NOS) activity, and *in vivo* NOS expression are also elevated in cortical CD11b^+^ microglia from wild-type mice upon acute d-METH exposure. Additionally, CD11b^+^ cells are the only ones found to exhibit changes in sleep-regulatory IL-1β expression in response to d-METH (Wang et al., 2014).	The effects of d-METH on sleep and wakefulness could be mediated partly by microglia, through exacerbated oxidative stress and pro-inflammatory cytokine release (Wang et al., 2014).
	**Narcolepsy**	
	Increased levels of IL-6 and TNFα are measured in the sera or plasma from narcoleptic patients (Cartier et al., 2005; Eltayeb et al., 2007; Dauvilliers et al., 2014).	Decrease in CCR expression could lead to a defect in the recognition and phagocytosis of damaged cells by microglia and consequently to a delayed resolution of acute inflammation. These defects could lead to enhanced autoimmunity resulting in the loss of hypocretin neurons.
	Reduced levels of microglia/macrophage-derived CCR1 and CCR3 are measured in peripheral blood samples from narcolepsy patients (Mignot et al., 1995; Tafti et al., 1996; Partinen et al., 2014).	
**Antigen presentation**		
Microglia can present antigens to T cells upon their binding to MHC class II expressed on their surface.	An increased microglial expression of MHC class II is measured in the central nervous system of narcoleptic dogs (Tafti, 2009).	Local infusion of a low-dose of the endotoxin lipopolysaccharide in rats, as a model of chronic inflammation, induces the loss of hypocretin neurons and increases the number of MHC class II-positive microglia in the lateral hypothalamus. Microglia-mediated inflammation might be a trigger for the loss of hypocretin neurons during narcolepsy (Maurovich-Horvat et al., 2014).
**Phagocytosis**		
Microglia prune synapses in a complement-dependent manner in contexts of health and disease.	A distinctive complotype, i.e., a combination of polymorphisms defining complement activity: BfS, C4A3, and C4B1, was identified in narcopleptic patients (Savill et al., 2002).	Exacerbation of complement-dependent microglial phagocytic activity is a plausible mechanism leading to the loss of hypocretin neurons.
	**Obstructive sleep apnea**	
	***Sleep fragmentation***	
	Microglial co-localization with the marker of glutamatergic axon terminals VGLUT1 is increased after 5 days of chronic sleep deprivation in mouse prefrontal cortex (Bellesi et al., 2017). In parallel, the total length of microglial process arborization, and the proportion of the microglial population showing less ramified morphologies, are significantly reduced (Bellesi et al., 2017).	These observations suggest an exacerbated microglial pruning of synapes, which could be mediated by the classical complement pathway considering that expression of the complement protein C3 was concomitantly increased by sleep deprivation (Bellesi et al., 2017).
**Immune surveillance**	***Chronic intermittent hypoxia***	
Microglia detect changes in homeostasis through their recognition of exogenous or endogenous danger signals, such as danger-associated molecular patterns (DAMPs).	Elevated levels of DAMPs are measured in blood samples from obstructive sleep apnea patients (Sapin et al., 2015), as well as in the hippocampus of rodent models of chronic intermittent hypoxia (Kiernan et al., 2016).	Microglia could respond to DAMPs induced by enhanced neuronal activity or psychological stress. The consequences on neuronal health and cognitive function are still undetermined however.

### Infection-induced sleep gain

“Sickness” is defined as the conjunction of adaptive changes that are elicited by activation of the immune system. Among those changes, infections induce stereotypic alterations in sleep activity. In rodents, sleep is divided into (1) non-rapid eye-movement (NREM) sleep [a state of deep sleep that is characterized by slow electroencephalogram (EEG) delta (0.5–4 Hz) waves] and (2) rapid eye-movement (REM) sleep (displaying electrical oscillations typical of brain activation but with inhibition of muscle tone and involuntary saccadic eye movements). During sickness, the ratio of NREM to REM sleep is increased (i.e., time in NREM sleep is increased while time in REM sleep is reduced, Lancel et al., 1995; Kapas et al., 1998; Mullington et al., 2000). A significant increase in the power of slow wave activity (SWA) is also observed (Kapas et al., 1998; Mullington et al., 2000; Majde and Krueger, 2005). SWA is commonly referred to as slow oscillation (Steriade et al., 1993a,b,c; Cowan and Wilson, 1994) and considered a physiological measure of the homeostatic drive for sleep (Borbely, 1982, 2001; Franken et al., 2001). Numerous clinical reports concur that both acute and chronic infections, as well as inflammatory diseases, are associated with sleep-related symptoms such as reduced sleep quality and increased fatigue (for review see Rohleder et al., 2012).

Many pathogens comprising gram-positive and gram-negative bacteria (Toth and Krueger, 1988, 1989; Krueger and Majde, 1994), viruses (influenza virus, rhinovirus, and human immunodeficiency virus) (Norman et al., 1988, 1990; Toth et al., 1995; Opp et al., 1996; Gemma and Opp, 1999), fungal organisms that include *Candida albicans* and the protozoan *Trypanosoma brucei brucei* (Kent et al., 1988; Toth and Krueger, 1989; Toth et al., 1994) induce a sleep response (for review, Krueger and Opp, 2016). Infectious agents do so via PAMPs that enhance the systemic and central production of pro-inflammatory cytokines, such as interleukin (IL)-1 and tumor necrosis factor-alpha (TNFα) (Imeri and Opp, 2009; Besedovsky et al., 2012; Krueger and Opp, 2016). IL-1 and TNFα are well-established sleep-regulatory substances that regulate NREM sleep duration and intensity by modulating neuronal activity in the hypothalamic preoptic area (Obal and Krueger, 2003), locus coeruleus (De Sarro et al., 1997), dorsal raphe nucleus (Manfridi et al., 2003), and cerebral cortex (Yoshida et al., 2004; Churchill et al., 2008). Hence, these cytokines act both at the circuit level and locally in the cortex and brainstem to promote sleep (Krueger, 2008). All these findings have been extensively reviewed (Krueger, 2008; Krueger et al., 2011; Zielinski et al., 2013; Krueger and Opp, 2016).

Microglia are the main producers of cytokines within the nervous system during inflammatory diseases (Renno et al., 1995; Van Dam et al., 1995; Buttini et al., 1997; Medana et al., 1997; Gregersen et al., 2000; Kettenmann et al., 2011; Delpech et al., 2015), indicating that these cells could play a crucial role in infection-induced sleep alterations. The effects of cytokines on sleep–wake behavior involve communication between neurons and microglia. As microglial processes constantly survey synaptic elements in a neuronal activity-dependent manner (Davalos et al., 2005; Nimmerjahn et al., 2005; Wake et al., 2009; Tremblay et al., 2010; Hristovska and Pascual, 2015), a recent publication hypothesized that pro-inflammatory cytokines may exert their somnogenic effects by promoting microglial attraction to synapses (Karrer et al., 2015). In line with this assumption, the authors found that TNFα induces neuronal production of the chemokines (**chemo**attractant cyto**kines**) CCL2, CCL7, and CXCL10, which bind to their receptors expressed by microglia and promote microglial process extension (Karrer et al., 2015). TNFα additionally upregulates neuronal Homer1a (Karrer et al., 2015), which was shown to drive the homeostatic scaling-down of excitatory synapses during sleep (Diering et al., 2017). According to the synaptic homeostasis hypothesis, information processing and decision-making during wakefulness drive strengthening of synapses, which is counterbalanced during sleep by a global weakening (de Vivo et al., 2017). Mice with a deletion of Homer1a also show reduced wakefulness with increased NREM sleep during the dark period (Naidoo et al., 2012). While it has yet to be demonstrated *in vivo*, these data provide a provocative mechanism by which TNFα could regulate the sleep-wake cycle.

Interestingly, microglia were recently revealed also to follow a circadian rhythm for protein expression that is controlled by their intrinsic molecular clock (Hayashi et al., 2013). This notably affects their production of cytokines (Fonken et al., 2015), and could not only alter their response to infectious agents, but also their influence on sleep.

Overall, the literature suggest microglia play direct (structural interactions with synapses) as well as indirect (release of inflammatory cytokines) roles in infection-induced sleep gain.

### Narcolepsy

Narcolepsy is characterized by excessive daytime sleepiness, sleep/wake fragmentation, hallucinations, sleep paralysis, and disturbed nocturnal sleep. The prevalence of this chronic sleep disorder ranges between 0.02 and 0.05% of the general population, with first symptoms appearing around 20–30 years of age, preferentially in males. After one or more years of progression, the disease stabilizes (Siegel, 1999; Longstreth et al., 2007). Narcolepsy is caused by a specific loss of hypothalamic hypocretin-producing neurons (85–95% of cells in humans), which coincides with low hypocretin levels in patients' cerebrospinal fluid (CSF) (Nishino et al., 2000; Thannickal et al., 2000; Mignot et al., 2002). Hypocretin knockout mice recapitulate human disease and are commonly used as an animal model of narcolepsy (Chemelli et al., 1999; Hara et al., 2001; Tabuchi et al., 2014). However, it remains unclear how hypocretin neurons are lost in the human pathophysiology.

Both genetic heritability and environmental triggers were found to be involved in disease progression. The cause and pathogenesis of narcolepsy are still unclear but likely involve the immune system. Human narcolepsy is closely associated (>95% of cases) with human leukocyte antigen (HLA) class II alleles in the major histocompatibility (MHC) region (Langdon et al., 1984; Matsuki et al., 1992; Mignot et al., 1994; Tafti, 2009), a classical hallmark of autoimmune diseases. Yet, its classification as an autoimmune disease is still a matter of debate (for review, see Mignot et al., 1995; Partinen et al., 2014).

Accumulating evidence allows us to consider a possible microglial implication in narcolepsy. Increased microglial expression of MHC class II (a hallmark of chronic activation) has been reported in the central nervous system (CNS) of narcoleptic dogs. Reactive microglia were predominantly located in the brainstem (including the reticular formation), basal forebrain, and amygdala (Tafti et al., 1996). A recent publication also reported reduced levels of the chemokine receptors CCR1 and CCR3 in peripheral blood samples of patients with narcolepsy (Cartier et al., 2005; Eltayeb et al., 2007; Toyoda et al., 2015). These receptors are expressed by microglia (in the brain) and other macrophages (in the bloodstream and other tissues) to ensure proper and coordinated inflammatory responses. It is hypothesized that a decrease in CCR expression leads to a defect in the recognition and phagocytosis of damaged cells by microglia and consequently to a delayed resolution of acute inflammation. These defects could lead to enhanced autoimmunity resulting in the loss of hypocretin neurons.

Clinical studies have also revealed that narcolepsy patients have subtly dysregulated cytokine levels in their serum and CSF consistent with markers of microglial reactivity (Okun et al., 2004; Dauvilliers et al., 2014; Maurovich-Horvat et al., 2014; Tanaka et al., 2014). Multiple independent studies that include different ethnic populations showed a preponderant increase in IL-6 and TNFα expression in sera or plasma from patients with narcolepsy (Okun et al., 2004; Maurovich-Horvat et al., 2014; Tanaka et al., 2014). Other studies have identified changes in IL-4 (Dauvilliers et al., 2014) or IL-8 (Tanaka et al., 2014) levels. To further assess the role of inflammation in the degeneration of hypocretin neurons, Gerashchenko et al. (Gerashchenko and Shiromani, 2004) infused a low-dose of the endotoxin lipopolysaccharide (LPS) in the lateral hypothalamus of rats as a model of local chronic inflammation. This induced a decline in the number of neurons, including hypocretin neurons, in the lateral hypothalamus. Chronic LPS infusion also increased the number of MHC class II-positive microglia in the lateral hypothalamus. These data suggest that microglia-mediated inflammation might be a trigger for the loss of hypocretin neurons during narcolepsy (Gerashchenko and Shiromani, 2004).

In addition to their important roles in inflammation and immune response, microglia are the main phagocytes of the brain (Sierra et al., 2013). Microglial phagocytosis is a pivotal mechanism for the clearance of cellular elements in the CNS, as demonstrated by their ability to engulf brain-specific cargo, such as axonal and myelin debris or apoptotic neurons. Additionally, recent data has shown that microglia can execute neuronal death by phagocytosing stressed-but-viable neurons, a process termed “phagoptosis” (Brown and Neher, 2014). Microglia are equipped with a complementary array of receptors enabling them to recognize their targets (the so-called “eat-me” signals) including proteins of the classical complement cascade (Savill et al., 2002; Ravichandran, 2010). Interestingly, a clinical study reported a correlation between narcolepsy and complement receptors (Cuccia et al., 1991). The thirty narcoleptic patients studied had a distinctive complotype, i.e., a combination of polymorphisms defining complement activity: BfS, C4A3, and C4B1 (Cuccia et al., 1991). One could hypothesize that exacerbation of complement-dependent microglial phagocytic activity is a plausible mechanism leading to the loss of (hypocretin) neurons as also observed in other neurodegenerative conditions such as Alzheimer's disease (Hong et al., 2016).

Overall, this evidence indicates that the homeostatic function of microglia is altered in the pathogenesis of narcolepsy, leading to an increased release of inflammatory factors, and alteration of their phagocytic activity. Both events alter microglia-neuron interactions and might support the degeneration of hypocretin neurons and subsequent sleep alterations.

### Obstructive sleep apnea: sleep fragmentation and chronic intermittent hypoxia

Obstructive sleep apnea (OSA), often associated with obesity, is a multifactorial systemic disease affecting approximately 10–25% of the general population worldwide (Peppard et al., 2013; Heinzer et al., 2015; Senaratna et al., 2017). In OSA the upper airways are narrowed or collapsed during sleep causing repetitive pauses of breathing (apneas) and concomitant reductions of blood oxygen level. Apneas are followed by increased breathing efforts, which in turn lead to episodic increases in blood carbon dioxide concentration. These events cause repeated arousals from sleep (sleep fragmentation) and/or excessive daytime sleepiness. OSA is often accompanied by neurocognitive dysfunction leading to impairments in attention and vigilance, learning, and memory, as well as executive functions (Zhou et al., 2016). If untreated, OSA associates with several comorbidities (Vijayan, 2012) such as cardiovascular disease (Sanchez-de-la-Torre et al., 2013) and metabolic syndrome, including obesity (Shechter, 2016) and insulin resistance (Ip et al., 2002), as well as mild cognitive impairment/dementia (Yaffe et al., 2011, 2014). Recent epidemiological studies also indicated that OSA may be associated with an increased risk of cancer (Nieto et al., 2012; Palamaner Subash Shantha et al., 2015; Gozal et al., 2016).

The two major acute pathophysiological consequences of OSA are: (1) tissue oxygen shortage/hypoxia with rapid re-oxygenation (i.e., chronic intermittent hypoxia, CIH) and (2) sleep fragmentation (SF) (Young et al., 1993). In OSA, most tissues, including the brain, are repeatedly exposed to reduced oxygen levels or a total lack of oxygen followed by rapid increases in oxygen availability. This pattern of repeated hypoxia-re-oxygenation (HRO) increases tissue levels of reactive oxygen species/reactive nitrogen species (ROS/RNS) and concomitantly activates the immune response as measured by the production of pro-inflammatory cytokines (Lavie, 2003, 2015). However, these cellular responses are not uniform across all tissues and vary with the severity of OSA.

In OSA patients, the poor health outcomes are a consequence of complex interactions between CIH and SF. However, with animal models it has been possible to begin dissecting the individual pathophysiological contributions of SF and tissue oxygenation. In the next sections we review each of them separately in light of a hypothesized microglial contribution.

### Chronic intermittent hypoxia

Because the HRO processes in OSA are similar to the ischemia-reperfusion events in a wide range of brain pathologies, data from these disease models provide valuable information regarding the cellular signaling pathways which are possibly involved. However, it should be noted that in OSA, the tissue exposures to hypoxia are cyclic, and most importantly chronic, and therefore the cellular responses can differ substantially from those of acute exposures (Almendros et al., 2014). The main trigger of the inflammatory response measured in CIH is oxidative stress (Wang et al., 2010), defined as an imbalance between pro-oxidant and anti-oxidant systems, resulting in an excessive production of ROS such as superoxide, hydrogen peroxide, and various RNS (Lavie, 2015). ROS damage critical cellular biomolecules thus leading to cell injury or death. In OSA models of CIH, signs of injured cells have been reported in various brain areas but the hippocampus and cerebral cortex appear to be most sensitive (Gozal et al., 2001; Xu et al., 2004). These lesions were proposed to contribute to the widespread neurocognitive defects encountered in OSA (Lim and Veasey, 2010; Harper et al., 2013).

Evidence of microglial phenotypic transformation such as microglia-specific inflammatory gene expression as well as morphological changes have been reported in CIH models of OSA (Smith et al., 2013; Sapin et al., 2015). A recent review (Kiernan et al., 2016) posits several putative mechanisms by which microglial physiological functions, which are now recognized to be crucial for plasticity, learning, and memory, may be affected by CIH pathophysiology:

- By ROS. Either directly (although this hypothesis was not yet confirmed) or indirectly, by local signals generated by ROS injured/dying neurons.
- By peripheral inflammation, a condition well established in OSA (Unnikrishnan et al., 2015). Signals of inflammation could reach microglia via neural transmission of vagal afferents reacting to circulating pro-inflammatory agents or the pro-inflammatory agents themselves could cross the blood-brain barrier to directly affect microglia.
- By responding to DAMPs such as HSP60, HMGB1, and MRP8/14. Elevated levels of DAMPs have been reported in blood samples from OSA patients (Wu et al., 2010), as well as in the hippocampus of rodent models of CIH (Gozal et al., 2002).

### Sleep fragmentation

There is a growing literature indicating that short or insufficient sleep is associated with low grade inflammation (Everson, 2005; Mullington et al., 2010; Aho et al., 2013) as well as cellular stress (Naidoo, 2012; Hakim et al., 2015). Recently, findings from longitudinal follow-up studies in patients with idiopathic REM sleep behavior disorder also revealed that most patients eventually develop neurodegenerative disorder, particularly Parkinson's disease or other synucleopathies (Stokholm et al., 2017). Positron emission tomography (PET scan) studies using the radiotracer [^11^C](*R*)-PK11195, which labels “translocator protein 18 kDa (TSPO),” mainly expressed by microglia (Banati, 2002) and considered to modulate their inflammatory activity and phagocytosis (Karlstetter et al., 2014), revealed an increased binding of the radiotracer in the *substantia nigra* of patients with idiopathic REM sleep behavior disorder, thus identifying microglial “activation” as a potential therapeutic target for halting or delaying the neurodegenerative process.

Two experimental studies in rodent models of chronic sleep deprivation have also reported microglial morphological changes that comprise the enlargement of their cell body (Hsu et al., 2003) and increased expression of pro-inflammatory cytokines (Wisor et al., 2011a). Very recently, Bellesi et al. revealed using confocal microscopy analysis that microglial homeostatic surveillance of the parenchyma is reduced after 5 days of chronic sleep deprivation in mouse prefrontal cortex. The total length of microglial process arborization and the proportion of microglial cells showing ramified morphologies were significantly reduced. These changes were accompanied by an increased microglial co-localization with the marker of glutamatergic axon terminals VGLUT1, suggesting exacerbated phagocytosis, which could be mediated by the classical complement pathway considering that expression of the complement protein C3 targeting synapses for elimination (Schafer et al., 2012) was concomitantly increased by sleep deprivation (Bellesi et al., 2017). Expression of the TAM receptor MERTK, which regulates microglial surveillance of the parenchyma and phagocytic behavior under physiological conditions (Fourgeaud et al., 2016), was also found to be increased by sleep deprivation (Bellesi et al., 2017). Interestingly, astrocytes were additionally shown in Bellesi et al., using high-resolution serial block-face scanning electron microscopy, to phagocytose synaptic elements, mainly presynaptic axon terminals, both after acute and chronic sleep deprivation (Bellesi et al., 2017). Glial cells reactivity thus appears to be implicated in the detrimental consequences of sleep loss, through neuronal circuit remodeling or loss of their essential physiological functions. Synaptic loss is considered the best pathological correlate of cognitive decline across a variety of conditions that include aging and neurodegenerative diseases (Duman and Aghajanian, 2012; Spires-Jones and Hyman, 2014).

In the case of OSA, however, the main sleep phenotype is SF (rather than sleep loss) coupled with an excessive daytime sleepiness and widespread cognitive dysfunction. In the last decade rodent models of SF have demonstrated that SF alone (without CIH) induces sleepiness and cognitive impairment (McCoy et al., 2007; McKenna et al., 2007; Tansey et al., 2007; Tartar et al., 2010; Ramesh et al., 2012), obesity (Wang et al., 2014), immune mediated metabolic dysfunction (Zhang et al., 2014), cardiovascular alterations (Carreras et al., 2014), and alterations of the gut microbiota (Poroyko et al., 2016). All of these consequences have an oxidative stress-mediated inflammatory component that could directly or indirectly affect microglial functions as proposed for CIH above. Gut microbiota can also directly influence microglial maturation, leading for instance to an attenuated production of pro-inflammatory cytokines upon immune stimulation (Erny et al., 2015). In addition, SF mediated increase in microglial surveillance of synaptic elements or remodeling of neuronal circuits could underlie some of the reported impairments in cognitive performance through synaptic loss.

### Recreational-drug induced sleep loss

Recreational drugs have deleterious consequences on sleep, with dextro-methamphetamine (d-METH) exerting its sustained wake-promoting effects (for up to several days) by elevating the monoaminergic tone. It is rarely prescribed due to concerns involving neurotoxicity, notably to dopaminergic and serotoninergic neurons, in addition to its aphrodisiac and euphoriant effects, among other mind-altering properties, and strong addictiveness. PET scans of chronic d-METH self-administrating individuals using the radiotracer [^11^C](*R*)-PK11195 revealed exacerbated microglial reactivity across several brain regions that receive dopaminergic or serotoninergic innervation, including the midbrain, striatum, thalamus, orbitofrontal, and insular cortices (Sekine et al., 2008). This microglial transformation is likely plastic and reverts to normal over longer periods of abstinence, since the binding levels of [^11^C](*R*)-PK11195 correlated inversely with the duration of withdrawal in d-METH abusers (Sekine et al., 2008).

The effects of d-METH on sleep and wakefulness could be mediated by microglia, through exacerbated oxidative stress and pro-inflammatory cytokine release (Wisor et al., 2011b). In mice undergoing microglial depletion [through delivery of ganciclovir in transgenics expressing the suicide agent herpes thymidine kinase under control of the CD11b promoter], the duration of daily wakefulness produced by d-METH was reduced by nearly 1 h. *Ex vivo* nitric oxide synthase (NOS) activity, and *in vivo* NOS expression were also elevated in cortical CD11b^+^ microglia from wild-type mice upon acute d-METH exposure. Additionally, CD11b^+^ cells, which mainly comprise microglia in the brain, were the only ones among the cerebral cortex found to exhibit changes in sleep-regulatory IL-1β pro-inflammatory cytokine expression in response to d-METH (Wisor et al., 2011b). In a follow up study by the same group, it was further shown that IL-1 receptor(R) deficiency potentiates, although modestly, the wake-promoting effects of d-METH (Schmidt and Wisor, 2012). The increased time spent in NREM sleep subsequent to d-METH-induced wakefulness was abolished in IL-1R knockout mice, while the increased time spent asleep after 3 h of behaviorally enforced wakefulness was similarly prevented in the knockouts. These findings indicate that microglial IL-1β signaling through IL-1R contributes to the hypersomnolence that ensues sleep loss, whether it is triggered pharmacologically by recreational drugs or through behaviorally-induced sleep deprivation (Schmidt and Wisor, 2012).

## Conclusion and perspectives

While microglial function was not a primary focus in several of the studies covered in this review, their overall findings indicate that microglial dysfunction (in the form of exacerbated phagocytic activity and neuroinflammation) may contribute to the pathophysiology of sleep disorders. These activities would be exerted in concert with other brain cells, vascular, glial, or neuronal, and the peripheral immune cells transiting to the brain. In light of these combined findings, substantial amount of evidence point toward an emerging role for neuroinflammatory processes in the pathophysiology of sleep disorders. Microglial (and astrocytic) phagocytosis of synaptic elements was also found to be exacerbated in rodent models of sleep deprivation, indicating their possible implication in its detrimental consequences on cognition. Microglia thus emerge as an important subject of investigation for future sleep-related studies, as already recognized in a variety of neuropsychiatric and neurodegenerative contexts. In addition to the diseases discussed in this review, it would be interesting to study the roles of microglia in the regulation of sleep functions: their contribution to memory consolidation and transformation (Dudai et al., 2015), synaptic homeostasis which downscales synapses during sleep to ensure learning during wake (Tononi and Cirelli, 2014), and the brain clearance from its toxic metabolites during sleep, in cooperation with astrocytes, which form a system of perivascular tunnels named the “glymphatics” (Xie et al., 2013).

## Author contributions

AN, HW, and MET contributed to the design, documentation, and writing of this review article.

### Conflict of interest statement

The authors declare that the research was conducted in the absence of any commercial or financial relationships that could be construed as a potential conflict of interest.
